# Stretch‐induced blood pressure moderation: A potential basis for the sense of well‐being accompanying stretching of upper back and neck muscles

**DOI:** 10.14814/phy2.70569

**Published:** 2025-09-16

**Authors:** Jorge L. Reyes, Ciana Keller, Marinos Kosmopoulos, David G. Benditt

**Affiliations:** ^1^ Cardiovascular Division, Department of Medicine University of Minnesota Medical School Minneapolis Minnesota USA; ^2^ Department of Medicine University of Minnesota Medical School Minneapolis Minnesota USA; ^3^ Department of Medicine, Division of Cardiology Johns Hopkins University School of Medicine Baltimore Maryland USA

**Keywords:** hypotension, muscle stretch, reflex syncope, stretch syncope, well‐being

## Abstract

Voluntary stretching of upper back and shoulder muscles is often associated with a sense of well‐being of unknown cause. The goal of this study was to examine the impact of shoulder/upper back stretching on heart rate (HR) and blood pressure (BP) responses in healthy individuals. Twenty‐four healthy individuals underwent continuous beat‐to‐beat HR and BP monitoring during active standing (AS) and during shoulder/upper back extension stretching. Measurements were compared using appropriate statistical tests. With AS, HR increased (median 24 bpm) and systolic BP decreased (median − 28 mmHg). Shoulder/upper back stretching elicited a similar BP drop but a lesser HR increment (*p* < 0.001). The HR increase per mmHg BP fall (∆HR/∆BP) was significantly lower during stretch than during AS (0.34 vs. 1.1 beats/min/mmHg; *p* < 0.001). Thus, the HR response with stretch‐induced BP fall averaged only 30.9% of that seen with AS. Shoulder/upper back muscle stretching induces transient BP reduction with only limited compensatory tachycardia compared to hypotension during AS. These findings suggest a neural reflex mechanism possibly initiated by muscle mechanoreceptors, with predominant vasodepression that may contribute to relaxation and a sense of well‐being.

## 1 INTRODUCTION

Upper back and shoulder stretching often contributes to a sense of well‐being and stress reduction. However, the neurophysiological basis for this phenomenon is incompletely understood (Beneka et al., 2024; Carlson & Curran, 1994). While traditional explanations have focused on mechanical factors such as improved flexibility and reduced muscle tension, there is a potential role for interoception—the sensing of internal bodily states—in mediating these relaxation effects. In this setting, interoceptive pathways may transmit information about the physiological state of large muscle groups (e.g., proprioception, status of stretch) to central neural structures involved in both autonomic regulation and conscious perception of bodily sensations (Craig, 2003; Garfinkel & Critchley, 2013). The relationship between this interoceptive signaling and the cardiovascular responses to stretching may provide insight into the neurobiological basis for stretch‐induced feelings of relaxation and well‐being.

In certain regions of the body (e.g., forearm, thighs, and legs), muscle activation has been shown to trigger (possibly initiated by muscle mechanoreceptors) sympathetic activation with subsequent increases in heart rate (HR) and blood pressure (BP) (Matsukawa et al., 1994; Murphy et al., 2011; Scherrer et al., 1990). However, such an effect would not be expected to favor a sense of well‐being. Consequently, the basis for the apparently beneficial relaxation effects of muscle activation by stretching the shoulders and upper back remains only incompletely explained.

The autonomic response to muscle stretching involves specialized mechanoreceptors (sometimes termed “ergo‐receptors”) located within skeletal muscle tissue (Scott et al., 2000). These receptors detect changes in muscle length and tension that are believed to be transmitted centrally, and ultimately modulate both sympathetic and parasympathetic neural outflow presumably via the spinal cord and central nervous system. This presumptive neural feedback system likely plays a critical role in cardiovascular homeostasis during various physical activities, yet its presence and potential physiological role remain poorly characterized.

Upper body stretching maneuvers are commonly performed during daily activities, including upon waking, during work breaks, or during exercise routines. While generally considered benign, these movements occasionally trigger lightheadedness or syncope in susceptible individuals (Mazzuca & Thomas, 2007; Mercante et al., 2024; Pelekanos et al., 1990; Sturzenegger et al., 1995; Villamar et al., 2022; Yeom et al., 2011). Understanding the physiological basis of these responses in healthy individuals may provide insight into the more pronounced reactions seen in those with stretch‐induced syncope.

This study examined the cardiovascular effects of shoulder and upper back stretching in healthy individuals, with particular focus on the relationship between BP changes and compensatory HR responses. By comparing the magnitude and direction of stretch‐induced BP and HR change with those observed during the first 20 s of active standing (AS), we aimed to ascertain whether a neural reflex mechanism, possibly triggered by ergo‐receptors in shoulder and upper back muscle groups, may be responsible for stretch‐induced transient BP reduction, which, if not excessive, may foster a sensation of relaxation and diminished stress.

## 2 METHODS

### 2.1 Study population

Twenty‐four otherwise healthy individuals referred to the University of Minnesota Medical Center for evaluation of suspected reflex syncope/near‐syncope due to causes unrelated to stretching served as the study population. Patients presented to the clinical laboratory in the morning and had been instructed not to take any cardioactive medications for at least 5 half‐lives before evaluation. This study was conducted in accordance with the Declaration of Helsinki (2013 revision) and was approved by the Institutional Review Board of the University of Minnesota Medical Center (Study Number: STUDY00020691). This report adheres to the COPE (Committee on Publication Ethics) guidelines. Written informed consent was obtained from all participants prior to enrollment.

### 2.2 Monitoring and procedures

Electrocardiographic leads were placed for continuous recording of HR. Blood pressure was continuously recorded using finger plethysmography (V‐1000, Edwards Life Sciences, Irvine, California). All subjects underwent comprehensive autonomic evaluation, which among the standard test panel included hemodynamic assessment during active standing, Valsalva maneuver, and respiratory sinus arrhythmia.

For the stretch maneuver, subjects were seated and asked to perform an extension maneuver targeting the neck and shoulder/upper back muscles. To isolate the effects of shoulder and upper back muscles, subjects were asked to keep their arms and hands immobilized to facilitate stable plethysmography recordings and to breathe normally throughout the test. A physician demonstrated the maneuver in each case prior to testing. To minimize “central command” interference, subjects were not alerted to when the stretch maneuver was to begin.

The specific maneuver consisted of asking patients to stretch their shoulder and upper back muscles by performing a shoulder elevation and exorotation while simultaneously extending their neck backward approximately 15–20 degrees. The shoulder elevation was performed to the maximum comfortable range without straining, typically reaching ear level. Subjects were instructed to hold this combined position for the duration of the maneuver, which lasted approximately 10–15 s. The maneuver was standardized by having the same physician demonstrate and supervise each test, ensuring consistent execution across all participants. BP and HR during the stretching maneuver were compared with measurements immediately before stretching.

In all study subjects, the relative impact of stretch on delta HR/delta BP was compared with delta HR/delta BP observed using the nadir BP usually with 10–13 s after active standing in the same individual. This comparison, termed stretch‐induced “chronotropic impact,” was calculated as a percentage: [(stretch value/active standing value) × 100].

### 2.3 Statistical analysis

Continuous variables were compared using the *t*‐test for parametric analysis and the Wilcoxon test for nonparametric analysis. The analysis was performed using R (R Foundation for Statistical Computing, Vienna, Austria).

## 3 RESULTS

The study comprised 24 young individuals (21 female, 3 male) with a median age of 33 years who were undergoing autonomic testing for suspected reflex syncope unrelated to stretching and who were otherwise in good health. During the shoulder/upper back stretch maneuver, all subjects exhibited a measurable decrease in BP from baseline (Table 1).

**TABLE 1:** Demographics and autonomic findings in study population.

Parameter	Healthy individual (*N* = 24)
Age (years)	34 (22,46.3)
Sex (female/male)	21/3
History of hypertension (%)	12.5
History of diabetes (%)	16.7
Resting SBP	135.5 (118.5, 143.5)
Resting MAP	100.8 (86.7, 104.3)
Resting HR	84 (77.0, 94.3)
Active standing nadir SBP	111 (97, 114)
Active standing nadir HR	114 (98.5, 123)
Delta active standing SBP	‐28 (−40, −16)
Delta active standing HR	24 (18.5, 35)
Delta HR/delta SBP active standing	−1.1 (−1.5, −0.5)
Near‐syncope symptoms while AS (%)	33.3
Valsalva ratio	1.50 (1.30, 1.96)
Abnormal Valsalva (%)	33.3
Respiratory sinus arrhythmia (bpm)	9.3 (6.6, 13.2)
Positive CSM	Not tested
Resting SBP before stretch	132.5 (117, 145.3)
Resting MAP before stretch	99.7 (91.7, 107.4)
Resting HR before stretch	89 (82, 100.8)
Time to nadir (s)	11 (7.7, 14.5)
Time to recovery (s)	8.6 (7, 10.2)
Stretch nadir SBP	98.5 (90.0, 118.8)
Stretch nadir MAP	79.2 (68.5, 90.6)
Stretch HR at BP nadir	100 (89.8, 107)
Delta SBP	−28.5 (−40.3, −15.0)
Delta MAP	−18.7 (−29.6, −8.3)
Delta HR	+9.5 (5.8, 14)
Delta HR/delta SBP stretch	−0.34 (−0.69, −0.18)
Syncope symptoms during stretching (%)	4.2

*Note*: Baseline demographic, autonomic and stretch testing characteristics of healthy individual. Blood pressure was measured in mm Hg and heart rate was measured in beats per minute. Categoric variables are expressed as percentages, and numeric variables as median (25, 75 quantiles).

Abbreviations: bpm, beats per minute; HR, heart rate; MAP, mean arterial pressure; SBP, systolic blood pressure.

The pre‐stretch values (SBP: 132.5 mm Hg [IQR 117‐145.3]; MAP: 99.7 mm Hg [IQR 91.7‐107.4]) dropped significantly during the maneuver, reaching nadir values approximately 11 s after starting the stretch (SBP: 98.5 mm Hg [IQR 90.0–118.8]; MAP: 79.2 mm Hg [IQR 68.5–90.6]; *p* < 0.001 for both comparisons) (Table 2).

**TABLE 2:** Statistical comparison of cardiovascular parameters between active standing and stretch maneuvers.

Parameter	*p*‐value
Delta HR (active standing vs. stretch)	0.001
Delta SBP (active standing vs. stretch)	0.966
Delta HR/delta SBP (active standing vs. stretch)	<0.001

This stretch‐induced hypotension was accompanied by a modest compensatory HR increase from an average of 89 beats/min (IQR 82–100.8) to 100 beats/min (IQR 89.8–107). BP typically recovered within 8.6 s after reaching nadir. Five participants (33.3%) reported lightheadedness during the BP decrease, and one experienced syncope or near‐syncope.

**FIGURE 1:**
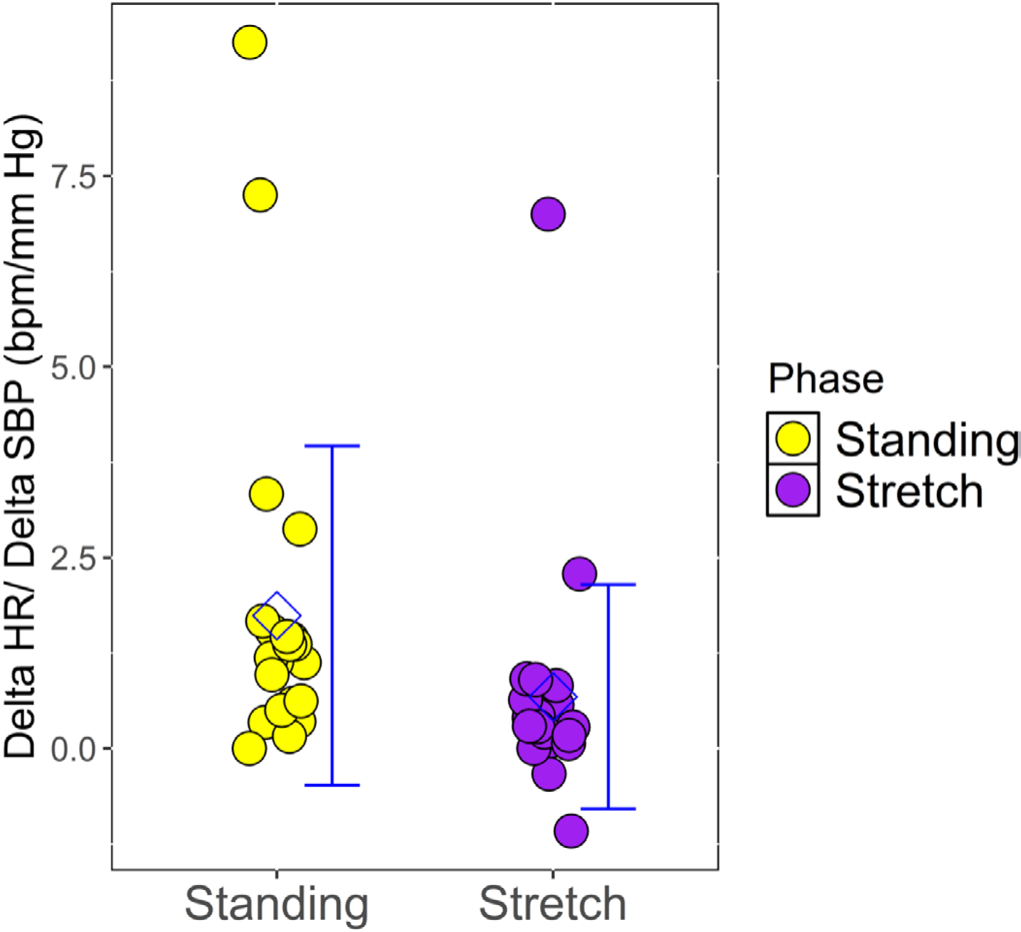
Scatter plot comparing the ratio of heart rate change to blood pressure change (ΔHR/ΔSBP) during active standing versus upper back and shoulder stretching in healthy individuals (*n* = 24). Each point represents an individual subject. Note the significantly lower ΔHR/ΔSBP ratio during stretching (mean −0.34 beats/min/mmHg) compared to active standing (mean −1.1 beats/min/mmHg; *p* < 0.001), indicating a blunted chronotropic response to similar magnitudes of blood pressure reduction.

Notably, when comparing cardiovascular responses between the stretch maneuver and AS for similar magnitudes of BP reduction, the HR responses were markedly different. With AS, the median SBP decreased by −28 mm Hg (IQR −40 to −16) to a nadir of 111 mm Hg (IQR 97–114), while HR increased by 24 beats/min (IQR 18.5–35) to 114 beats/min (IQR 98.5–123). On the other hand, despite a similar BP fall during stretching (SBP −28.5 mm Hg [IQR −40.3 to −15.0]), HR increased by only 9.5 beats/min (IQR 5.8–14) (Figure 1).

The ratio of heart rate change to systolic BP change (ΔHR/ΔSBP) during AS, averaged −1.1 beats/min/mm Hg (IQR −1.5 to −0.5), reflecting a presumably normal baroreflex‐mediated compensatory tachycardia response to hypotension in these otherwise healthy subjects. In contrast, the same ratio during stretching was only −0.34 beats/min/mm Hg (IQR −0.69 to −0.18), or approximately 30.9% of the active standing response (*p* < 0.001).

**FIGURE 2:**
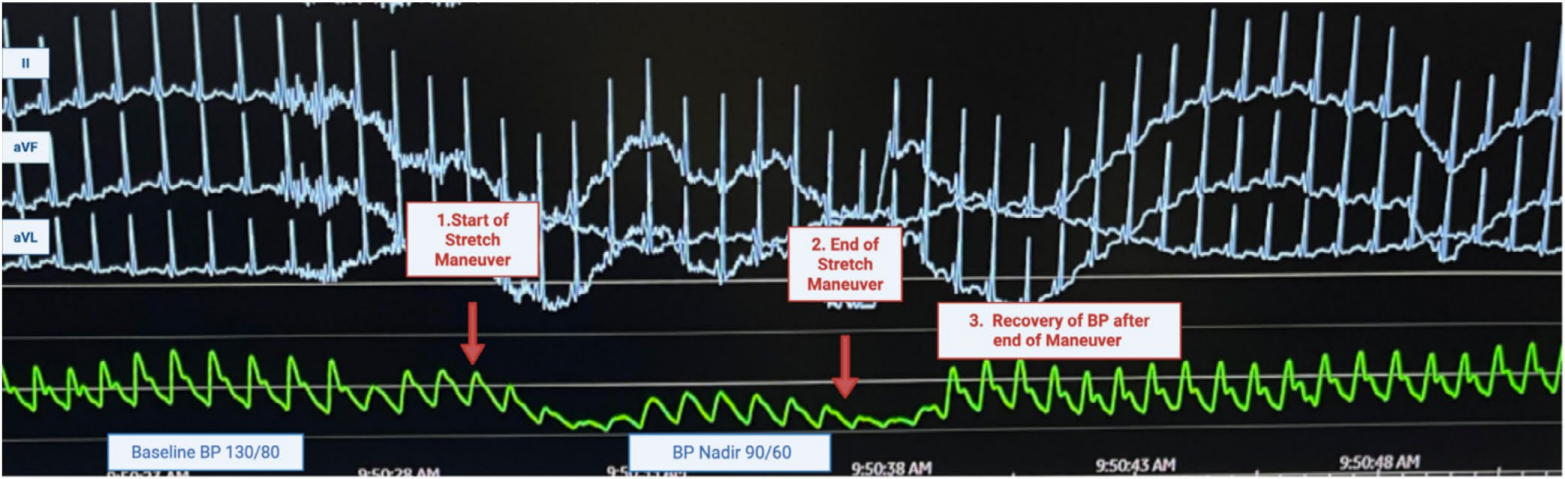
Baseline blood pressure of 140/90 prior to initiation of stretch maneuver with resulting drop in BP to 98/57 by end of stretch maneuver with slow recovery of blood pressure back to baseline. Leads aVR, aVL, aVF, and V1 seen as above.

Thus, while the magnitude of BP decrease was nearly identical between stretch and AS (*p* = 0.966), the compensatory HR response during stretching was significantly attenuated compared to active standing (*p* = 0.001) (Figure 2).

## 4 DISCUSSION

Our findings reveal that shoulder and upper back stretching produces previously unappreciated but potentially clinically important cardiovascular effects in healthy individuals. Specifically, the observed hypotensive response with shoulder and neck stretching occurs in conjunction with a distinctly blunted chronotropic response compared to the transient immediate HR increase observed in the same individual during orthostatic challenge (i.e., active standing, AS). This pronounced asymmetry between vasodepression and chronotropic compensation suggests the involvement of a neural reflex mechanism initiated at the level of the activated muscle group.

### 4.1 Physiology of stretch‐induced cardiovascular changes

The physiological basis for the upper back and shoulder stretch induced phenomenon likely involves a complex interplay between muscle mechanoreceptors and central autonomic control centers. Shoulder and upper back stretching may activate specific populations of muscle afferents that simultaneously trigger peripheral vasodilation while modulating central cardiovascular control. Previous research has demonstrated that passive stretching in the back muscles, triceps, and gastrocnemius and soleus muscle can elicit parasympathetic activation with concurrent sympathetic inhibition (Kosmopoulos et al., 2025; Trajano et al., 2020), consistent with our observations of hypotension accompanied by limited compensatory tachycardia.

Several neural pathways could potentially mediate the apparent vasodepression and relative cardio‐inhibition effects. Muscle mechanoreceptors, including muscle spindles and Golgi tendon organs, transmit afferent signals that modulate autonomic outflow at multiple levels. Recent studies have identified specialized stretch‐sensitive ion channels (such as PIEZO channels) that respond to mechanical deformation and may play roles in peripheral vascular control (Li et al., 2014; Wang et al., 2016). Additionally, central integration of these signals during stretch coming from various mechanoreceptors in a variety of possible originating muscle beds may differ from that associated with traditional orthostatic challenges, potentially explaining the unique pattern of chronotropic restraint we observed.

The relationship between the observed cardiovascular responses and subjective well‐being likely involves interoceptive mechanisms. Interoception—the brain's perception of internal bodily states—plays a crucial role in emotional experience and stress regulation. (Craig, 2003; Garfinkel & Critchley, 2013) The distinctive pattern of hypotension with limited compensatory tachycardia we observed during stretching may create a specific interoceptive signal that the brain interprets as relaxation or reduced stress. Unlike the interoceptive signature of orthostatic stress (rapid tachycardia with hypotension) generally associated with the large muscle groups of the lower extremities, which is typically perceived as physiologically threatening, the neck/shoulder stretch‐induced pattern may be interpreted as physiologically non‐threatening with lesser need for aggressive HR increment despite similar drops in blood pressure. This differential interoceptive processing could explain why upper back and shoulder stretching produces feelings of relaxation rather than distress, despite causing measurable cardiovascular changes. Recent neuroimaging studies have shown that pleasant interoceptive stimuli activate brain regions associated with reward processing (Khoury et al., 2018), suggesting a potential neural pathway through which stretch‐induced cardiovascular changes might generate positive affective states.

While future studies should indeed incorporate validated well‐being measures, our primary contribution in this report is identifying the potential physiological basis for a phenomenon that has been empirically observed but mechanistically unexplained. The cardiovascular signature we discovered provides a measurable, reproducible correlate that may underlie the subjective experience of relaxation associated with upper back stretching.

### 4.2 Clinical implications

Shoulder and upper back stretching induces a transient hypotensive response in healthy individuals. Importantly, the compensatory HR increment associated with stretch‐induced hypotension is significantly less than would be expected for comparable BP fall during active standing, suggesting chronotropic restraint.

From a clinical perspective, these findings have implications beyond understanding stretch‐induced syncope. The hypotensive effect of upper body stretching may offer therapeutic applications for individuals with hypertension (Ko et al., 2021; Yamada et al., 2022). Conversely, patients with orthostatic intolerance or autonomic disorders should be cautious when performing upper body stretching maneuvers, particularly in upright positions where the hypotensive effects could compound orthostatic stress. Furthermore, the distinctive chronotropic profile observed during stretching might serve as a useful diagnostic tool for assessing autonomic function.

In comparing our findings with previous research, we note both similarities and differences with other forms of reflex‐mediated cardiovascular responses. Unlike vasovagal syncope, which typically involves pronounced bradycardia alongside hypotension (Jardine et al., 2018), the stretch response features hypotension with limited tachycardia (i.e., relative cardioinhibition) rather than bradycardia. This pattern distinguishes stretch‐induced hypotension from classic vasovagal mechanisms while still suggesting neural reflex involvement.

### 4.3 Future research directions

The findings presented here suggest several promising avenues for future investigation. Several potential studies are summarized. First, a few reports indicate that some patients seem to have a compulsion to trigger the muscle stretching, suggesting the possibility that the effect is pleasurable. Controlled studies incorporating validated well‐being measures (e.g., Visual Analog Scales for relaxation, Positive and Negative Affect Schedule) could quantitatively correlate the stretch effect in terms of pleasure and relaxation responses. The use of back massage of differing intensity may permit reproducible assessment. Second, controlled assessment of sympathetic tone changes during stretch can be undertaken by invasive measurement of muscle sympathetic nerve activity via microneurography or indirectly by assessing heart rate variability changes during stretch. Direct measurement is invasive and requires expertise but is feasible. Indirect heart rate variability assessment is constrained by the duration of time that stretching or massage is carried out. However, both are feasible with readily available technology. Third, examination of age and sex differences in stretch‐induced cardiovascular responses is a reasonable next step as our patient population to date is predominantly young females. Finally, preliminary findings suggest that the muscle stretch triggers a spinal cord reflex that we believe is modulated by the central nervous system. In most cases, the outcome is tolerable hypotension. However, we hypothesize that inadequate central nervous system control may be responsible for certain patients developing more severe hypotension, occasionally leading to lightheadedness or even syncope. Studies using certain short‐term pharmacological central nervous system stimulants could be undertaken, and the stretch responses compared to placebo in the same individuals. Those patients who do manifest marked hypotension with stretch, although few in numbers, are severely impacted in terms of adverse effects on lifestyle. Consequently, many would be willing to undertake such studies, assuming the pharmacologic intervention is safe. In brief, targeted investigations such as these could transform our preliminary observations into evidence‐based therapeutic applications while enhancing our understanding of the neurophysiological basis for interoceptive well‐being.

### 4.4 Strengths and limitations

This study has several notable strengths that support its validity and importance. We utilized continuous beat‐to‐beat monitoring of both HR and BP, providing high temporal resolution data that captured the rapid cardiovascular changes occurring during the stretch maneuver. The study design included a within‐subject comparison of stretch responses to active standing, allowing us to control for individual variations in autonomic responsiveness. All participants underwent comprehensive autonomic testing, including the Valsalva maneuver and respiratory sinus arrhythmia, providing important context for interpreting their stretch responses. The standardized protocol for the stretch maneuver, with direct supervision and consistent verbal instructions, ensured reproducibility across participants. Furthermore, by studying individuals without stretch‐related syncope, we were able to characterize the normal physiological response to upper back stretching without the confounding influence of pathological responses.

Nevertheless, some limitations warrant consideration. Our sample size was small and predominantly consisted of relatively young female participants referred for evaluation of syncope. The latter may limit generalizability to broader populations. We did not directly measure muscle activity or tension during the stretching maneuver, which could have provided additional insights into the relationship between muscle activation and cardiovascular responses. While our findings strongly suggest a neural reflex mechanism by virtue of the observation of relative cardio‐inhibition during stretch‐induced hypotension, we did not perform neuroimaging or direct measures of sympathetic and parasympathetic activity, which limits our ability to definitively characterize the neural pathways involved.

## 5 CONCLUSION

Shoulder and upper back stretching induces a transient immediate BP fall in healthy individuals, but the expected accompanying HR increase is significantly less than would be expected for comparable BP fall during an orthostatic maneuver such as active standing, suggesting chronotropic restraint. The distinctive interoceptive signature created by this physiological pattern may explain how stretching of the upper back and shoulders promotes a sense of well‐being, as the brain interprets these particular cardiovascular changes as signals of safety, and the resulting moderate BP drop and modest HR increment favor relaxation rather than physiological threat. Our findings further support the upper back stretch‐induced muscle activation operating via a neural reflex mechanism involving both vasodepression and limited compensatory tachycardia. While not proven, it seems reasonable to hypothesize that the reflex is triggered through activation of muscle mechanoreceptors, and the reflex provides a potential physiological basis for the widely reported sense of well‐being that accompanies upper back and shoulder stretching.

## CONFLICTS OF INTEREST STATEMENT

All authors meet authorship criteria and all have approved the manuscript for publication. The authors have no conflicts of interest.

## ETHICS STATEMENT

The study was approved by the Institutional Review Board of the University of Minnesota Medical School. This report adheres to the COPE (Committee on Publication Ethics) guidelines. Written informed consent was obtained from all participants prior to enrollment.

## Data Availability

The datasets generated and analyzed during the current study are available from the corresponding author upon reasonable request, in compliance with institutional privacy policies and applicable regulations.
